# Anthocyanins From *Clitoria ternatea* Flower: Biosynthesis, Extraction, Stability, Antioxidant Activity, and Applications

**DOI:** 10.3389/fpls.2021.792303

**Published:** 2021-12-17

**Authors:** Gayan Chandrajith Vidana Gamage, Yau Yan Lim, Wee Sim Choo

**Affiliations:** School of Science, Monash University Malaysia, Subang Jaya, Malaysia

**Keywords:** blue pea, blue colourant, delphinidin, functional food, genipin, phycocyanin, spirulina, ternatin

## Abstract

*Clitoria ternatea* plant is commonly grown as an ornamental plant and possesses great medicinal value. Its flower is edible and also known as blue pea or butterfly pea flower. The unique feature of anthocyanins present in blue pea flowers is the high abundance of polyacylated anthocyanins known as ternatins. Ternatins are polyacylated derivatives of delphinidin 3,3′,5′-triglucoside. This review covers the biosynthesis, extraction, stability, antioxidant activity, and applications of anthocyanins from *Clitoria ternatea* flower. Hot water extraction of dried or fresh petals of blue pea flower could be employed successfully to extract anthocyanins from blue pea flower for food application. Blue pea flower anthocyanins showed good thermal and storage stability, but less photostability. Blue pea flower anthocyanins also showed an intense blue colour in acidic pH between pH 3.2 to pH 5.2. Blue pea flower anthocyanin extracts demonstrate significant *in vitro* and cellular antioxidant activities. Blue pea flower anthocyanins could be used as a blue food colourant in acidic and neutral foods. The incorporation of blue pea flower anthocyanins in food increased the functional properties of food such as antioxidant and antimicrobial properties. Blue pea flower anthocyanins have also been used in intelligent packaging. A comparison of blue pea flower anthocyanins with two other natural blue colouring agents used in the food industry, spirulina or phycocyanin and genipin-derived pigments is also covered. Anthocyanins from blue pea flowers are promising natural blue food colouring agent.

## Highlights

– Blue pea flower contains high amount of blue colour anthocyanins.
– Blue pea flower contains polyacylated anthocyanins called ternatins.
– Blue pea anthocyanins demonstrate good thermal and storage stability.
– Anthocyanins from blue pea flower is a good alternative to spirulina and genipin.

## Introduction

Food colourants play an important role in food industry altering or conferring colours to food to increase the customer attractiveness and sensory acceptability (Lin et al., 2018). Food colourants are classified as artificial and natural, based on their origin. Artificial food colours are chemicals which originate from coal tar derivatives, and most of them contain an azo group (Dilrukshi et al., 2019). Considering artificial blue colours, Brilliant Blue FCF (E133, FD&C Blue No. 1) and Indigo Carmine/Indigotine (E132, FD&C blue No. 2) are approved as food colours in the European Union and the United States. Patent Blue V (E131) is authorised as a food additive in the European Union (Directive 94/36/EC; US Food and Drug Administration (FDA, 2015). Artificial blue colourants are used in various types of food. A study done in the Iranian market found that Brilliant blue colourant was commonly found in edible ices, jelly, fruit drink powder, chocolate/ice cream powder, soft drink, syrup, and candy (Asadnejad et al., 2018). Natural food colours consist of pigments such as anthocyanins, carotenoids, chlorophyll etc. that are extracted from mainly plants and micro-organisms (Sen et al., 2019). The demand for food products with natural colouring agents has increased since consumption of synthetic colourings are believed to cause allergies, food intolerance, hyperactivity, irritability and sleep disorders in children (Feketea and Tsabouri, 2017). Pigments giving red, orange and yellow hues are widely available but only a few sources are available giving blue colour. Anthocyanins are present in fruits giving rise to blue colour (Choo, 2019). Apart from anthocyanins, commonly used blue colourants in the food industry are spirulina/phycocyanin (a protein extracted from cyanobacteria *Spirulina platensis*, from eukaryote algae such as Rhodophytes and Cryptophytes and *Galdieria sulphuraria*, a unicellular rhodophyte) and the blue colour pigments produced by the reaction between primary amines and genipin (a colourless iridoid from monoterpenes class extracted from fruits of both *Genipa americana* and *Gardenia jasminoides* Ellis) (Landim Neves et al., 2021). Both spirulina and the blue pigment derived from genipin possess both advantages and disadvantages, that are unique to them. For example, phycocyanin is stable in the presence of citric acid, sugar, and soluble in warm or cold water but being a protein, it tends to get denatured in elevated temperature, low pH and is highly unstable under light (Gustiningtyas et al., 2020). Genipin-derived pigments show good thermal, photostability but become unstable in the presence of ascorbic acid. From an industrial perspective, the extraction procedures to obtain blue pigments from both spirulina and genipin are complicated. The extraction of spirulina involves the chemical breakdown of cell walls of respective organisms and the production of genipin-derived pigments involves a synthesis step from its precursor (Buchweitz, 2016). Therefore, new sources of natural blue colour are needed.

Anthocyanins are the largest group of water-soluble pigments belonging to flavonoids, a subclass of the polyphenol family, contributing to the attractive orange, red, purple, violet, and blue colours of fruits, vegetables, and flowers (Jing and Giusti, 2007). More than 700 anthocyanins have been identified in nature and they play a vital role in pollination and protecting plant cells from ultra-violet (UV) radiation (Salehi et al., 2020). Anthocyanins are glycosides of anthocyanidins. Pelargonidin, cyanidin, peonidin, delphinidin, petunidin and malvidin are the most common anthocyanidins in the plant kingdom (Choo, 2019). Colour of the anthocyanins depend on the pH of the solution because the structure of the anthocyanins alters depending on the pH of the surrounding medium (Khoo et al., 2017). Anthocyanins have been used in traditional medicine and for colouring food since ancient times. The therapeutic effects of anthocyanins are mainly attributed to their antioxidant activities (Khoo et al., 2017). The structure of anthocyanins allows anthocyanins to display direct antioxidant activity toward radicals in two mechanisms named: hydrogen atom transfer (HAT) and single electron transfer (SET). In both mechanisms, the anthocyanin becomes a free radical itself, but it is more stable and the oxidative damage from the initial free radical is prevented (Garcia and Blesso, 2021). Anthocyanins have demonstrated several other health benefits such as antibacterial, antiproliferative, hypoglycaemic etc. (Yoon et al., 2018; Li et al., 2019; Yue et al., 2019). Therefore, the application of anthocyanins as food colourants should be encouraged to deliver these health benefits to consumers. Generally, anthocyanins are well known for their unstable nature since the stability of anthocyanins is influenced by factors such as chemical structure, pH, temperature, light, presence of oxygen, solvent, the presence of co-pigments, metal ions, and enzymes (Vidana Gamage et al., 2021a). Blue colour anthocyanins are generally found in blue colour flowers and fruits. *Clitoria ternatea* flower is one anthocyanin source containing stable blue colour polyacylated anthocyanins (Abidin et al., 2019; Thuy et al., 2021). The presence of polyacylated anthocyanins, metal ions, other phenolic compounds and the resulting co-pigmentation effect may assist to form more stable and intense blue colours (Yoshida et al., 2009).

*Clitoria ternatea* L. commonly known as butterfly pea or blue pea is a perennial leguminous herb belonging to family Fabaceae having several beneficial agricultural and medical applications, such as fodder, nitrogen-fixing crop, an eco-friendly insecticide (Oguis et al., 2019), food colouring (Pham et al., 2019), and in traditional medicine for disorders such as anasarca and ascites (Lakshan et al., 2019). It is commonly grown as an ornamental plant and is also used for revegetation (Kosai et al., 2015). Blue pea plants are distributed in several countries all over the world such as Thailand, Malaysia, Kenya, Australia, the United States, Sri Lanka, Brazil, Cuba, Sudan etc. (Havananda and Luengwilai, 2019). Blue pea flower is being eaten as vegetables in Southeast Asia (Leong et al., 2017) and blue pea flower extract has been used in desserts and beverages in Southeast Asian countries such as Malaysia and Thailand (Pasukamonset et al., 2017). Polyacylated derivatives of delphinidin 3,3′,5′-triglucoside, named “ternatins” are the major anthocyanins present in blue pea flower (Terahara et al., 1990; Vidana Gamage et al., 2021b). All ternatins carry the basic structure of delphinidin-3, 3′, 5′-triglucoside. A series of 15 ternatins A1-A3, B1-B4, C1-C5 and D1-D3 have been discovered so far (Nair et al., 2015; Jeyaraj et al., 2020). This review focus on the biosynthesis, extraction, stability, antioxidant activity and applications of anthocyanins from blue pea flower. Specifically, the potential of using blue pea flower anthocyanins as a natural blue food colouring agent is also covered.

## Blue Pea Flower Anthocyanins

**FIGURE 1:**
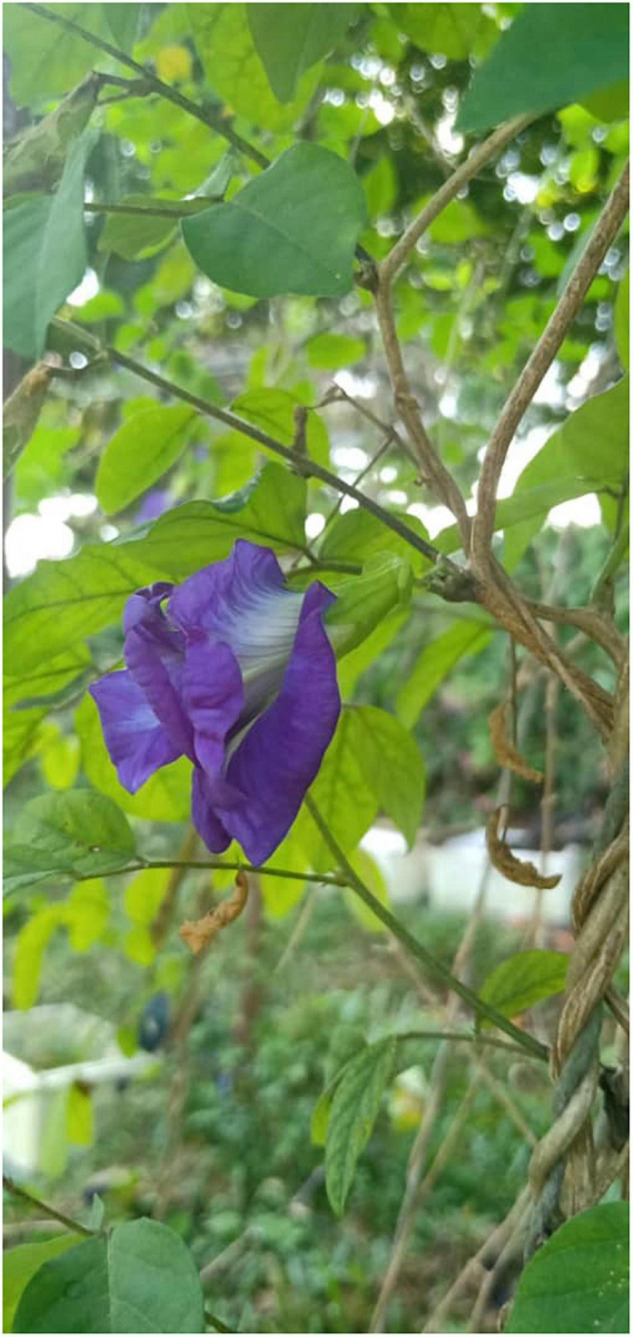
Blue pea flower (*Clitoria ternatea*).

**FIGURE 2:**
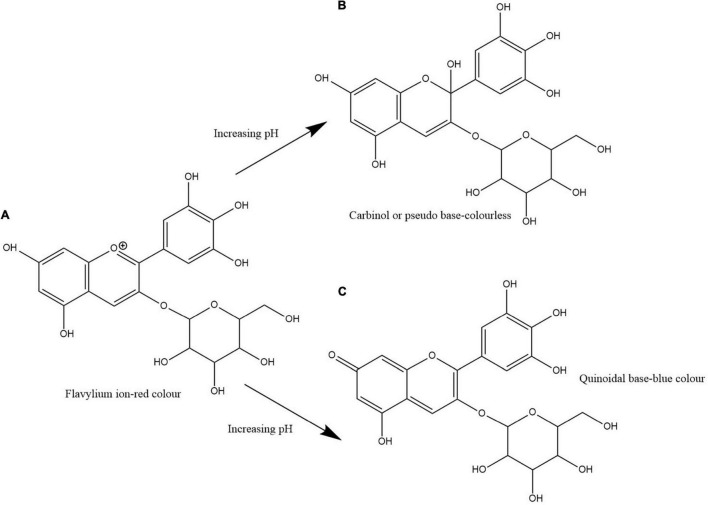
Structural change of delphinidin-3-glucoside with increasing pH. Path **(A,B)** shows the transformation of flavylium ion to carbinol or pseudo base. Path **(A,C)** shows the transformation of flavylium ion to quinoidal base. Path **(A,C)** shows the structural alteration responsible for the blue colour formation in blue pea flower anthocyanins.

*Clitoria ternatea* L./blue pea flower (Figure 1) is a rich source of polyacylated anthocyanins and their higher stability compared with non-acylated anthocyanins provide the advantage to be used as a natural food colouring agent (Buchweitz et al., 2012; Marpaung et al., 2019). Like all anthocyanins, the colour of blue pea flower anthocyanin extract also changes with pH. At pH lower than 3.2 the red colour exists, from pH 3.2 to 5.2 the colour changes from violet to blue, from pH 5.2 until pH 8.2 light blue colour exists and from pH 8.2 to pH 10.2 the colour changes from light blue to dark green colour (Escher et al., 2020a). This colour change could be explained by the structural alteration occurring in anthocyanin molecules along with the change in H^+^ and OH^–^ concentration in the medium. The red colour is attributed to the presence of flavylium ion, blue colour to the presence of the neutral quinoidal base and the green colour to ionic chalcone (Liu et al., 2014). In non-acylated anthocyanins, flavylium ion transforms to colourless carbinol pseudo base when pH increases. But in blue pea flower anthocyanins, the presence of acyl groups prevents the hydrolysis of flavylium ion to less stable carbinol pseudo base form and instead form the blue colour quinoidal that possess less sensitivity to pH changes in mildly acidic or neutral medium (Bridle and Timberlake, 1997). Therefore, blue pea flower anthocyanins could be used as a blue colouring agent in acidic and neutral food systems. Figure 2 shows the structural alteration of delphinidin-3-glucoside with increasing pH and formation of blue colour in blue pea flower anthocyanins.

## Biosynthesis of Ternatins

The anthocyanin biosynthetic pathway is an extension of the general flavonoid pathway (Tanaka et al., 2009). Anthocyanin biosynthesis pathway is an elucidated metabolic pathway involving enzymes such as chalcone synthase (CHS), chalcone isomerase (CHI), flavanone 3-hydroxylase (F3H), flavonoid 3′-hydroxylase (F3′H), flavonoid 3′,5′-hydroxylase (F3′5′H), dihydroflavonol 4-reductase (DFR), anthocyanidin synthase (ANS), glycosyltransferase (GT), and acyltransferase (AT). Anthocyanins are synthesised in the cytoplasm of the cell and then transported to the vacuole. The vacuolar transportation of anthocyanins takes place in several pathways that include endoplasmic reticulum-derived vesicles and a tonoplast-bound glutathione S-transferase-like transporter (Collings, 2019). However, acylation of anthocyanins, catalysed by acyltransferases (ATs) and the final modification of anthocyanins occurs after being transported to the vacuole (Lu et al., 2021).

**FIGURE 3:**
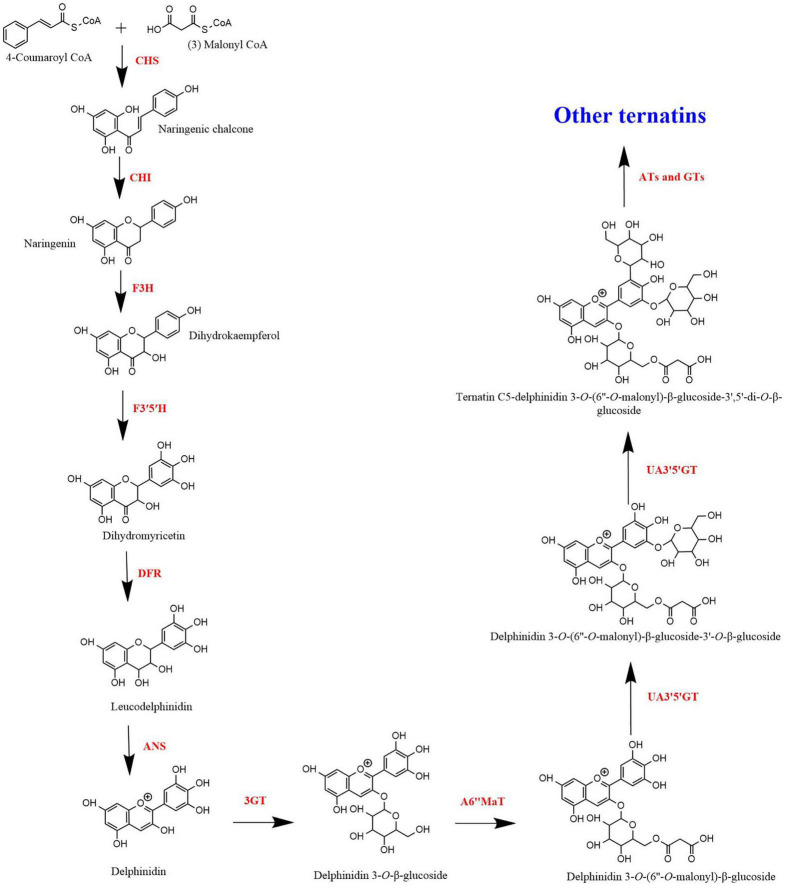
Biosynthesis of ternatins.

Figure 3 shows the proposed ternatin synthesis pathway drawn based on the studies done by Kogawa et al. (2007a) and Tanaka et al. (2009). In the biosynthesis of ternatins, first delphinidin-3-*O*-β-glucoside is formed and then it is modified by further glucosylation and acylation. For the synthesis of delphinidin-3-*O*-β-glucoside, 4-coumaroyl-CoA and malonyl-CoA act as precursors. Synthesis of the naringenin chalcone from the two precursors is mediated by CHS. CHS is the initial key enzyme of flavonoid biosynthesis. Then, naringenin chalcone is isomerised by CHI to naringenin. Naringenin is then converted to dihydrokaempferol in the presence F3H. Dihydrokaempferol is then converted to dihydromyricetin by F3′5′H. Both F3′H and F3′5′H are the enzymes responsible for the diversification of anthocyanins by determining their B-ring hydroxylation pattern and consequently the colour of the anthocyanins (Liu et al., 2018). Therefore, F3′5′H directly contribute to the blue colour anthocyanins in blue pea flower, since increased hydroxylation of the B-ring shifts the anthocyanin colour toward blue (Togami et al., 2006). Next, dihydromyricetin is converted into colourless leucodelphinidin mediated by DFR and subsequently to delphinidin by ANS. According to Kogawa et al. (2007a), a glucosyl group is added to delphinidin by anthocyanin 3-*O*-glucosyltransferase (3GT) to form delphinidin 3-*O*-β-glucoside. Kogawa et al. (2007a) stated that other glucosyl groups are added to the B-ring of delphinidin 3-*O*-β-glucoside, only after malonylation of delphinidin 3-*O*-β-glucoside. Accordingly, delphinidin 3-*O*-β-glucoside is malonylated in the presence of anthocyanidin 3-*O*-glucoside 6″-*O*-malonyltransferase (A6″MaT). Then, two glucose molecules are added to delphinidin 3-*O*-(6″-*O*-malonyl)-β-glucoside, first to 3′ position followed by 5′ position (Kazuma et al., 2004). This glycosylation is mediated by anthocyanin 3′,5′-*O*-glucosyltransferase (UA3′5′GT) in two subsequent steps (Kogawa et al., 2007b). The molecule is now referred to as delphinidin 3-*O*-(6″-*O*-malonyl)-β-glucoside-3′,5′-di-*O*-β-glucoside and can be called as ternatin C5. Ternatin C5 is the simplest ternatin. Other 14 ternatins are synthesised by adding acyl and glucosyl groups to ternatin C5 in the presence of acyltransferases (ATs) and glucosyltransferases (GTs). Acyltransferases (AT) also plays a major role resulting in the blue colour and the stability of ternatins, because polyacylation of ternatins with p-coumaroyl groups results in a shift of colour of anthocyanins to bluish region due to intramolecular co-pigmentation among acyl moieties and between acyl moieties and anthocyanin chromophore (Honda and Saito, 2002). Furthermore, polyacylation at the 3′ position of anthocyanins results in stable blue colouration (Lu et al., 2021). This is the main reason for the high stability of ternatins because most ternatins are polyacylated at the 3′ position. Therefore, when studying the ternatin biosynthesis pathway, hydroxylation, glycosylation and acylation can be considered as the most important steps that are responsible for synthesising stable blue colour anthocyanins in blue pea petals.

## Extraction of Blue Pea Flower Anthocyanins

Extraction is the first important step in the recovery of active ingredients from plant materials (Jeyaraj et al., 2020). The purpose of selecting a suitable extraction method is to obtain the maximum yield with a high concentration of target compounds. Since anthocyanins are sensitive to heat, light, acids and alkalis, selecting a suitable extraction method to get the maximum amount of anthocyanins without degradation is critical (Chandrasekhar et al., 2012; Jeyaraj et al., 2020).

Considering conventional solvent extraction methods, type of solvent, substrate: solvent ratio, extraction temperature, extraction time and soaking time may affect the extraction yield and total anthocyanin content (TAC) of a blue pea flower anthocyanin extract (Rocha et al., 2020). Selection of solvent should be done based on the application of the anthocyanin extract. Therefore, when anthocyanins from blue pea flowers are extracted for food application, the use of hazardous organic solvents should be avoided (Khoo et al., 2017; Chemat et al., 2019). Several studies have used hydro alcoholic extraction [i.e., 37% ethanol (Jaafar et al., 2020), 50% ethanol (Pham et al., 2019), 50% methanol (Shen et al., 2019)] to extract anthocyanins from blue pea flower. However, FDA (2018) has categorised methanol as a class 2 solvent having inherent toxicity and ethanol as a class 3 solvent that should be limited by good manufacturing practices (GMP) and other quality-based requirements. Distilled water is the best solvent for extracting anthocyanins for food applications because water could be considered as a non-toxic, non-flammable, and inexpensive green solvent (Chemat et al., 2019). Therefore, this review focuses only on the extraction of anthocyanins from blue pea flowers with water. Table 1 shows previous studies that carried out extraction of anthocyanins from blue pea flowers using water as the solvent.

**TABLE 1:** Previous studies on extraction of anthocyanins from blue pea flower using water and the antioxidant activity of extracts.

Extraction technique	Optimum extraction condition	Extraction yield	Anthocyanin content	Antioxidant activity	References
Water extraction of fresh petals	Substrate: solvent ratio – 1:20 (g/mL) Temperature –60°C Time – 60 min	56.1%	4.0 mg cyanidin-3-glucoside/g	IC_50 –_ 1.18 mg/mL in DPPH radical scavenging activity FRAP assay-19.8 mg of gallic acid equivalent/g of extract Inhibit reactive oxygen species in RAW264.7 cells by 75-80%	Jeyaraj et al., 2021
Water extraction of dried ground petals	Substrate: solvent ratio – 1:20 (g/mL) Temperature – 60°C Time – 60 min	–	32.6 mg cyanidin-3-glucoside/L	–	Ahmad et al., 2020
Water extraction of dried ground petals	Temperature – 90°C Time – 5 min	–	15.2 mg delphinidin-3-glucoside equivalent/g dried flower	–	Voss et al., 2020
Water extraction of dried ground petals	Substrate: solvent ratio – 1:10 (g/mL) Temperature – 100°C Time – 30 min	–	3.61 mg cyanidin-3-glucoside/g	IC_50 –_ 195.5 μg/mL in DPPH radical scavenging activity IC_50 –_ 42.9 μg/mL in ABTS free radical assay Protect human keratinocytes from H_2_O_2_-induced cytotoxicity	Zakaria et al., 2018
Water extraction of dried ground petals	Substrate: solvent ratio – 1:100 (g/mL) Temperature – 80°C Time – 30 min	–	8.67 mg ternatin B2 equivalent/g dried flower	467.04 μmol Trolox equivalent/g dried flower in ABTS assay	Vuong and Hongsprabhas, 2021
Water extraction of dried ground petals	Substrate: solvent ratio – 1:37 (g/mL) Temperature – 54°C Time – 74 min	45.5%	–	–	Baskaran et al., 2019
Water extraction of dried ground petals and spray dried	Substrate: solvent ratio – 1:20 (g/mL) Temperature – 90–95°C Time – 240 min	–	1.08 mg delphinidin-3-glucoside/g	–	Chusak et al., 2018
Water extraction of dried ground petals and spray dried	Substrate: solvent ratio – 1:6 (g/mL) Temperature – 100°C Time – 120 min	–	1.46 mg cyanidin-3-glucoside/g	IC_50_ – 0.47 mg/mL in DPPH radical scavenging activity ORAC value-17.54 μg trolox equivalents/mg dried extract Protected erythrocytes from APPH induced haemolysis and oxidation	Phrueksanan et al., 2014
Water extraction of dried ground petals and partial purification of lyophilised extract using Amberlite XAD7HP resin	Substrate: solvent ratio – 0.125:25 (g/mL) Temperature – 40°C Time – 30 min	2%	–	The aqueous extract showed 43–64% inhibition for DPPH radical scavenging assay	Escher et al., 2020a
Extraction of fresh petals with water at pH 1and 2	Substrate: solvent ratio – 1:3 (g/mL) Temperature – room temperature Maceration time – 24 h pH – 1	–	58.06 mg cyanidin-3-glucoside/L	–	Saptarini and Suryasaputra, 2018
Extraction of dried, ground petals soaking in distilled water	Substrate: solvent ratio − 1:20 (g/mL) Soaking time − 0 h Extraction temperature – 60°C Extraction time − 20 min	–	58.2 μg cyanidin-3-glucoside/mL	IC_50_ – 10.9 mM TE/g in DPPH radical scavenging activity Inhibited seven−keto cholesterol production in an emulsion of cholesterol and a free radical generator by 79.8%	Shen et al., 2019
Ultrasound-assisted water extraction of fresh petals	Substrate: solvent ratio − 1:15 (g/mL) Temperature − 50°C Time – 150 min Ultrasonic power – 240 W	–	1.12 mg delphinidin-3-glucoside/g	–	Gwee and Chong, 2015
Ultrasound-assisted water extraction of fresh petals	Substrate: solvent ratio − 1:50 (g/mL) Temperature − 60°C Time – 90 min Ultrasonic power – 100 W	–	1.42 g/L	–	Syafa’atullah et al., 2020
Ultrasound-assisted water extraction of fresh petals	Substrate: solvent ratio – 1:20 (g/mL) Temperature − room temperature Time – 60 min Ultrasonic power – 560 W	36.1%	4.2 mg cyanidin-3-glucoside/g	–	Jeyaraj et al., 2021
Ultrasound-assisted water extraction of dried ground petals	Substrate: solvent ratio – 1:10 (g/mL) Temperature − 40°C Time – 45 min Ultrasonic power – 160 W	–	1.77 mg cyanidin-3-glucoside/g	The aqueous extract showed 63.8% inhibition for DPPH radical scavenging assay	Salacheep et al., 2020
Microwave-assisted water extraction of dried petals	Substrate: solvent ratio − 1:20 (g/mL) Microwave power – 770 W Time – 1 min	–	30.9 mg cyanidin-3-glucoside/L	–	Marsin et al., 2020

Saptarini and Suryasaputra (2018) showed that the anthocyanin content of blue pea flower anthocyanin extract that was extracted using water at pH 1 was higher than that of pH 2. This shows that the pH of water used for extraction can affect the TAC of the blue pea flower anthocyanin extract. Kang et al. (2021) suggested that the reason for obtaining a higher extraction efficiency when using more acidic solvents is the higher stability shown by the anthocyanins in acidic medium. Some studies use a soaking step before extracting anthocyanins. However, Shen et al. (2019) showed that soaking is not necessary when extracting anthocyanins from blue pea flowers using water. Soaking significantly reduced the TAC of the blue pea flower anthocyanin extract from 58.2 to 39.9 μg cyanidin-3-glucoside/mL when the petals were soaked in water for 24 h but there was no significant difference in the TAC of the blue pea flower anthocyanin extract when soaking was carried out for 6 and 12 h. The reason for the reduction of TAC due to soaking could be attributed to the increase of hydrolysis of anthocyanins when more water molecules are available in the matrix (Matsufuji et al., 2007; Marpaung et al., 2017). Therefore, extraction of anthocyanins from blue pea flower with water could be done without a soaking step that gives an advantage in time factor during the extraction process.

Considering substrate: solvent ratio, 1:20 (g/mL) was reported by Chusak et al. (2018) and Ahmad et al. (2020) as the best ratio for extraction of anthocyanins from blue pea flower (Table 1). The volume of water used for extraction is an important factor because less solvent would not extract a sufficient amount of anthocyanins from blue pea petals and excess water requires more energy for evaporation. Temperature around 50–60°C and extraction time around 20–60 min could be considered as the most suitable extraction temperature and time for extraction of anthocyanins from blue pea flower (Table 1) because higher temperature and long extraction time may result in deterioration of anthocyanins (Loypimai et al., 2016; Aprodu et al., 2020). High temperature and long extraction durations are disadvantageous in terms of energy consumption. Extraction conditions also affect the antioxidant activity of the blue pea flower anthocyanin extract. Shen et al. (2019) showed that the antioxidant activity of a blue pea flower anthocyanin extract was dependent on the solvent used for extraction and the soaking time upon extraction. However, there was no significant difference in the 2,2diphenyl-1-picrylhydrazyl (DPPH) radical scavenging activity of blue pea flower anthocyanin extract that was extracted using distilled water without soaking (10.9 mM Trolox equivalents (TE)/g dry basis) and after soaking for 6 h (11.7 mM TE/g dry basis). Soaking in water up to 24 h significantly reduced the antioxidant activity of blue pea flower anthocyanin extracts (9.45 mM TE/g dry basis) extracted by distilled water (Shen et al., 2019). Furthermore, the antioxidant activity of blue pea flower anthocyanin extract that was extracted with 50% methanol (12.2 mM TE/g dry basis) was not significantly different in DPPH radical scavenging activity compared to blue pea flower anthocyanin extract that was extracted using distilled water (10.9 mM TE/g dry basis) at 0 h soaking. Similarly, Jeyaraj et al. (2021) also found that there was no significant difference in the antioxidant activity of the blue pea flower anthocyanin extracts obtained from water extraction and 50% ethanol extraction measured using DPPH and ferric reducing antioxidant power (FRAP) assays. This suggests that blue pea flower anthocyanins can be extracted using distilled water and used as a natural blue colouring agent with high antioxidant activity. Therefore, when extracting anthocyanins from blue pea flowers for food applications, hot water extraction with short extraction time is preferred.

Conventional solvent extraction techniques consume more solvents, time, thermal energy and are associated with several disadvantages. To overcome the shortcomings of conventional solvent extraction and to increase extraction efficiency, several non-conventional extraction techniques were explored. Ultrasonication is one such technique. In ultrasound-assisted extraction, acoustic cavitation causes molecular movement of solvent and sample results in the breakdown of plant cell walls and membranes and facilitate their movement to surrounding solvent (Chemat et al., 2011). One study employed ultrasound-assisted water extraction of blue pea flower anthocyanins and showed that ultrasound-assisted water extraction yielded 246.48% better anthocyanin extract yield compared with the conventional ethanolic extraction. According to that study, the TAC of the blue pea flower extract (1.126 mg delphinidin-3-*O*-glucoside equivalent/g) obtained by ultrasound-assisted water extraction was higher than that (0.325 mg delphinidin-3-*O*-glucoside equivalent/g) obtained by conventional ethanolic extraction (Gwee and Chong, 2015). Marsin et al. (2020) applied microwave-assisted extraction to extract blue pea flower anthocyanins, but the TAC of the blue pea flower anthocyanin extract obtained from microwave-assisted extraction did not show a higher value compared to the TAC of the blue pea flower anthocyanin extract obtained from conventional hot water extraction in the study by Ahmad et al. (2020) (Table 1). Therefore, the use of ultrasound-assisted extraction is promising to extract blue pea flower anthocyanins for food applications. When extracting anthocyanins from blue pea flower petals using water, other bioactive compounds that are soluble in water are also extracted together. Therefore, those compounds may also contribute to the antioxidant property of the anthocyanin extract and greater extraction efficiency could result in a higher antioxidant activity of the extract (Jeyaraj et al., 2020). The blue pea flower anthocyanin extract obtained by ultrasound-assisted extraction demonstrated 47.21% more DPPH radical scavenging activity compared to the blue pea flower anthocyanin extract obtained from conventional ethanolic extraction (Gwee and Chong, 2015). Further studies need to be carried on investigating the effect of other novel extraction technologies such as high-pressure processing, sub-critical water extraction etc. to extract blue pea flower anthocyanins.

## Stability of Anthocyanins From Blue Pea Flower

The stability of anthocyanins is highly affected by pH, temperature, light, metal ions in media etc. thus their application in food products is limited (Vidana Gamage et al., 2021a). Therefore, if anthocyanins are used as food colourants, those anthocyanins should possess reasonable thermal, photo and storage stabilities. This is because during food processing the food materials undergo several heat processes such as pasteurisation or sterilisation where else during storage, food must withstand storage conditions and photo stress (Charurungsipong et al., 2020). Several studies have investigated the stability of anthocyanins from blue pea flowers concerning their thermal, storage and photo stabilities.

Temperatures above 50°C cause partial or complete degradation of anthocyanins from natural sources and this results in a reduction of colour intensity (Escher et al., 2020a). Anthocyanins from blue pea flower demonstrate good heat stability in acidic pH. Blue pea flower anthocyanins at pH 3.6 and 5.4 showed stability at 60 and 70°C. The absorbance remained unchanged for 360 min but when the temperature was increased from 70 to 100°C, the degradation constant significantly increased. At pH 3.6 the degradation rate constant increased from 5.57 × 10^–4^ to 3.41 × 10^–3^min^–1^, when temperature increased from 80 to 100°C and at pH 5.4 the degradation rate constant increased from 5.33 × 10^–4^ to 3.32 × 10^–3^ min^–1^ when temperature increased from 80 to 100°C (Escher et al., 2020a). Another study supports the above finding that the degradation rate of blue pea flower anthocyanins increases when the temperature is above 70°C (Lee et al., 2011).

One study investigated the degradation of anthocyanins from blue pea flowers at 28, 60, and 90°C. The degradation half-lives at each temperature were 8.63, 5.16, and 3.75 min, respectively. This shows how the degradation rate increased (half-life reduced) with increasing temperature. This study also showed that the addition of catechin as a co-pigment to the anthocyanin extract of blue pea flower at pH 3.5 increased the half-life of anthocyanins from blue pea flower to 8.39 at 90°C (Charurungsipong et al., 2020). Catechin molecules also have a similar structure as anthocyanins where each molecule possesses two benzene rings and one dihydropyran heterocycle. A co-pigment complex is formed between catechin and ternatins by intermolecular co-pigmentation either as an interlock complex or parallel complex (Charurungsipong et al., 2020). The presence of several aromatic acyl groups in ternatins may increase the chance of forming several co-pigment complexes with the available co-pigments. Generally, most food products fall into the pH range of pH 3.6 to 5.4 and the pasteurising temperature also ranges between 60 and 70°C (Low-Temperature Long Time (LTLT) – 62.5°C for 30 min and High-Temperature Short Time (HTST) – 72°C for 15 seconds) (Ranieri et al., 2009). This stability makes anthocyanins from blue pea flowers suitable as a natural food colour in functional food. Another study investigated the storage stability of anthocyanins from blue pea flowers with different temperatures in terms of colour stability index (absorbance on sampling day/absorbance of day 0). This study showed that at frozen and refrigerated conditions the colour stability index of anthocyanins from blue pea flower reduced only by 0.2 points after 30 days of storage. At temperatures above 25°C, the stability of blue pea flower anthocyanins reduced significantly. Their colour stability index reduced by 0.8 points during 20 days of storage at 25°C. The storage stability of anthocyanins from blue pea flowers was significantly low when stored at temperatures above 50°C and the colour stability index reduced by 0.9 points in 15 days of storage at 50°C (Ab Rashid et al., 2021). Another study showed that, when anthocyanin extract from blue pea flower was stored at 5°C, 80% of initial anthocyanins were retained after 30 days and the residual colour remained almost unchanged for about one year and the stability reduced within a week when stored between 25 and 37°C but at 45°C again the anthocyanins from blue pea flower demonstrated good stability for more than 10 days (Lee et al., 2011). This could be due to the activation of enzymes that cause anthocyanin degradation since 37°C is the optimum temperature for enzyme activity (Daniel and Danson, 2013). Therefore, it is recommended to use anthocyanins from blue pea flowers for food stored in cold conditions (e.g., frozen desserts, yoghurt, cool drinks) and to store food containing anthocyanins from blue pea flowers in the freezer, refrigerator or temperatures below 25°C.

Anthocyanins from blue pea flowers are less stable to photo stress compared to thermal stress (Mahmad and Taha, 2018). When anthocyanins from blue pea flowers were exposed to the direct light of a white fluorescent lamp (20 W) at 32°C, the anthocyanins from blue pea flowers degraded at a higher rate compared with anthocyanins kept covered from light. Blue pea flower anthocyanins at pH 3.6 had a 34.4% retention percentage when exposed to light whereas the covered anthocyanins had a 90.1% retention percentage after equal periods. Blue pea flower anthocyanins at pH 5.4 had a 48.3% retention percentage when exposed to light whereas the covered anthocyanins had a 94.6% retention percentage after equal periods (Escher et al., 2020a). Anthocyanins from blue pea flower demonstrated higher photostability at pH 5.4 compared with 3.6 but in both cases, the degradation rate looks high. But microencapsulation of anthocyanins from blue pea flowers was able to increase the photostability of blue pea flower anthocyanins (Marsin et al., 2020; Ab Rashid et al., 2021). Marsin et al. (2020) optimised the microwave-assisted encapsulation of blue pea flower anthocyanins and obtained an encapsulation efficiency of 73.24% with 40% (w/w) maltodextrin at 770 W microwave power for 7 min. Ab Rashid et al. (2021) reported an encapsulation efficiency of 87.3% using spray drying as the encapsulation technique and 20% (w/w) maltodextrin as the carrier agent. When microencapsulated anthocyanins from blue pea flower with maltodextrin and control blue pea flower anthocyanins were exposed to light at 25°C and pH 5.5, microencapsulated anthocyanins from blue pea flower showed a significantly higher colour stability index (colour stability index – 0.83) compared to the control anthocyanin extract from blue pea flower (colour stability index – 0.57) after 21 days (Ab Rashid et al., 2021). Therefore, when manufacturing food with blue pea flower anthocyanins as a food colourant, it is recommended to avoid using transparent packaging material to protect anthocyanins from direct light exposure or use an encapsulation technique.

The good thermal stability up to 70°C, the storage stability at 25°C and the intense blue colour demonstrated at pH 3.6 to 5.4, makes anthocyanins from blue pea flowers, suitable to be used as a blue colour food colourant. Table 2 shows a comparison of extraction yield, thermal stability, photostability, antioxidant activity and aggregate formation in acidic beverages of natural blue colouring agents used in the food industry: phycocyanin, genipin-derived pigments and anthocyanins from blue pea flowers. The extraction yield on dry basis of blue pea flower anthocyanin extract was higher than that of phycocyanin and genipin (Table 2). One possible reason for obtaining a lower extraction yield could be the presence of structures such as hard cell walls and fibre in the sources of phycocyanin and genipin that hinders the extraction (Buchweitz, 2016). Considering pH stability, blue pea flower anthocyanins demonstrated higher stability in acidic pH compared to phycocyanin and genipin-derived pigments.

**TABLE 2:** Comparison of common blue food colourants with anthocyanins from blue pea flower.

	Phycocyanin	Genipin derived pigments	Anthocyanins from blue pea flower
Extraction yield (% on dry basis)	8.27–8.66% (Chaiklahan et al., 2018)	10.7–11.8% (Buchweitz, 2016)	21–29% (Mehmood et al., 2019)
Thermal stability	Unstable (40–75% colour loss) at pH 4 and 8 when heated at 65°C for 30 min. Stable below 45°C (Patel et al., 2004)	Stable at pH 9 (colour remaining >90%) compared with pH 5 and 7 between 60 and 90°C for 10 h (Paik et al., 2001)	Stable at 60 and 70°C between pH 3.6 and 5.4 for 360 min (Escher et al., 2020a)
Photostability	Stable at pH 5 for 5 days under natural light and ambient temperature, unstable at pH 3 and 7 (Zhang et al., 2021)	Stable (colour remaining >90%) at pH 7 compared with pH 5 and 9 for 10 h under 5,000 lux and at 4°C. Stability reduced when light intensity increased from 5,000 to 20,000 lux (Paik et al., 2001)	Unstable to light (Escher et al., 2020a)
Antioxidant activity	DPPH radical scavenging activity-42.02 g ascorbic acid equivalent/100 g (Benchikh et al., 2020)	ORAC – 231.1 μM Trolox equivalent/g (Neri-Numa et al., 2018)	DPPH radical scavenging activity-12.2 mM Trolox equivalent/g (Shen et al., 2019)
Aggregate formation in acidic beverages	Formation of aggregates in acidic beverages with pH around pH 3 (Zhang et al., 2020)	Unstable in acidic beverages with pH around pH 3 (Buchweitz, 2016)	No aggregate formation in acidic solutions with pH around pH 3 (Lakshan et al., 2019)

Comparing the thermal stability of phycocyanin, genipin-derived pigments and anthocyanins of blue pea flower (Table 2), phycocyanin was less stable at low-temperature long time pasteurisation conditions (at 63°C for 30 min) but Chaiklahan et al. (2012) reported that the colour loss of phycocyanin in high-temperature short-time pasteurisation (71°C for 15 s) was negligible. The thermal stability of genipin-derived pigments was higher in the alkaline medium compared with acidic and neutral media, but most of the blue coloured food products consist of an acidic pH (Asadnejad et al., 2018). Contrastingly, blue pea flower anthocyanins showed stability in acidic pH under pasteurising conditions (63°C for 30 min). Considering photostability, both phycocyanin and blue pea flower anthocyanins showed unstable nature against photo stress compared to genipin-derived pigments (Table 2). Therefore, special attention should be paid when using blue pea flower anthocyanins or genipin-derived pigments as a food colour to avoid photodegradation. Both phycocyanin and genipin-derived pigments were not stable in the acidic medium whereas the blue pea flower anthocyanins demonstrated high stability in acidic pH around pH 3 to 5. Products such as soft drinks, fruit drinks and jelly mainly contain artificial blue colourants belonging to this pH range (Asadnejad et al., 2018). This could be considered as a major advantage of using blue pea flower anthocyanins as a blue colourant over phycocyanin and genipin-derived pigments. All three blue colourants showed antioxidant activities, but a direct comparison cannot be made as they were determined by different antioxidant assays.

## Antioxidant Activity of Blue Pea Flower Anthocyanins

The antioxidant property is the ability to donate hydrogen atoms or electrons to free radicals and displace free radicals, thus preventing the damage caused by the free radicals (Tan and Lim, 2015). Anthocyanins demonstrate both *in vivo* and *in vitro* antioxidant activity (Migliorini et al., 2019; Vidana Gamage et al., 2021a). It is believed that blue pea flower anthocyanins could prevent cardiovascular and neurological diseases, cancer and diabetes, due to their antioxidant capabilities (Cazarolli et al., 2009; Shen et al., 2019). The toxicological safety of using blue pea flower extracts have been prove from some studies. An aqueous extract of blue pea flower petals showed no cytotoxicity in human fibroblast (IMR90) cells (LC_50_ > 900 μg/mL) and showed a protective effect in human erythrocytes and inhibited the oxidation of pBR322 plasmid DNA (Mehmood et al., 2019; Escher et al., 2020b). A water extract of blue pea flower was non-toxic up to 625 μg/mL on RAW264.7 cells (Jeyaraj et al., 2021). The *in vitro* antioxidant properties displayed by water extracts of blue pea flower anthocyanins are shown in Table 1.

In one study, it was found that blue pea flower anthocyanin extract demonstrated significant antioxidant activity against DPPH and peroxyl radicals. Using DPPH assay, the IC_50_ (concentration of the antioxidants needed to decrease the initial free radical concentration by 50%) of the blue pea flower anthocyanin extract (0.47 mg/mL) was significantly higher compared to that of ascorbic acid (0.002 mg/mL) (Phrueksanan et al., 2014). Similarly, in the study done by Zakaria et al. (2018), the anthocyanin extract from blue pea flower showed significant scavenging activity against DPPH and 2,2′-azino-bis (3-ethylbenzthiazoline-6-sulphonic acid (ABTS) radical scavenging assays (Table 1) and the IC_50_ values of the Trolox standard for both DPPH (IC_50_ – 3.32 μg/mL) and ABTS assays (IC_50_ – 6.51 μg/mL) were significantly lower compared to blue pea flower anthocyanin extract. This indicates that ascorbic acid and Trolox possess higher antioxidant activity compared with the blue pea flower anthocyanin extract.

Shen et al. (2019) also studied how the antioxidant property of blue pea flower anthocyanins prevent lipid oxidation. Lipid oxidation occurs when free radicals are available in the surrounding medium. A dose of 6 mg/mL blue pea flower anthocyanins extracted by distilled water was able to inhibit 7-keto cholesterol production in an emulsion of cholesterol and a free radical generator by 79.8% after 48 h of treatment (Shen et al., 2019). 7-keto cholesterol is formed from the cholesterol-free radical chain reaction through 7-hydroxyperoxycholesterol (7-OOH) dehydration or 7-hydroxycholesterol (7-OH) dehydrogenation (Xu et al., 2005). Therefore 7-keto cholesterol is used to measure the extent of lipid oxidation. Furthermore, the inhibition of lipid oxidation could be explained by the antioxidant activity of blue pea flower anthocyanins against free radicals. In another study, Jeyaraj et al. (2021) investigated the antioxidant activity of a water extract of blue pea petals, based on the ability of the blue pea flower extract to reduce the extent of 2,2′-azobis-2-methyl-propanimidamide dihydrochloride (AAPH)-generated free radicals in RAW264.7 cells (mouse macrophage cells). It was found that the water extract with a concentration of 156.3 μg/mL blue pea flower extract demonstrated 75-80% inhibition against AAPH-generated free radical formation. Likewise, Phrueksanan et al. (2014) found that anthocyanin-rich extract of blue pea petals could protect erythrocytes from AAPH-induced haemolysis and oxidative damage. It was found that an anthocyanin-rich extract of blue pea petals (400 μg/mL) could reduce membrane lipid peroxidation, protein carbonyl group formation and prevent the reduction of glutathione concentration in APPH induced haemolysis (Phrueksanan et al., 2014). Furthermore, Zakaria et al. (2018) showed that a blue pea flower anthocyanin extract could protect human keratinocytes from H_2_O_2_-induced cytotoxicity and UV-induced mtDNA damage in human keratinocytes. The cellular antioxidant activity of blue pea flower extract is clearly demonstrated in these studies. Overall, the results of *in vitro* antioxidant assays should not be interpreted directly as the antioxidant property inside the body of a living being (Gengatharan et al., 2015; Jeyaraj et al., 2020). Therefore, the antioxidant properties of blue pea flower anthocyanins in living systems should be investigated.

A major cause of non-communicable diseases is hyperglycaemia (Kim and Oh, 2013). Increased serum glucose levels after a meal could create several complications such as the production of mitochondrial reactive oxygen species (ROS) that can deplete antioxidant enzymes in serum. Studies have found that bioactive compounds such as anthocyanins could inhibit the action of carbohydrate digestive enzymes such as pancreatic α-amylase and intestinal α-glucosidase consequently reducing postprandial hyperglycaemia (McDougall et al., 2005). One study investigated the effect of ingestion of blue pea flower anthocyanins with or without sucrose on the glucose level and antioxidant capacity of the serum of humans. Eighteen healthy men between 18 and 40 years were selected and administered with different sucrose, water and blue pea flower anthocyanin extract treatments (1 or 2 g of blue pea flower anthocyanin extract + 400 mL water, 50 g sucrose + 400 mL water, 1 or 2 g of blue pea flower anthocyanin extract + 50 g sucrose + 400 mL water) after 12 h fasting period. Subjects who were administered with 1 and 2 g of blue pea flower anthocyanin extract with 400 mL of distilled water did not show any change in serum glucose level. It was observed that the subjects who ingested sucrose and water, had a rapid increase in plasma glucose level approximately by 75 mg/dL after 30 min of administration and fell back to normal level within 90 min. Subjects who were administered with 1 or 2 g of blue pea flower anthocyanins with 50 g sucrose and 400 mL distilled water demonstrated an increase of serum glucose level only by 60 mg/dL within 30 min of ingestion but reduced significantly within 60 min and came to normal level in 90 min. The postprandial plasma glucose concentration after 30 and 60 min of ingestion was significantly lower (*p* < 0.05) in the sucrose treatment with blue pea flower anthocyanins when compared to the sucrose treatment with water. Plasma insulin level increased in sucrose only treatment, but plasma insulin level did not change significantly with blue pea flower anthocyanins treatment. When sucrose was administered with blue pea flower anthocyanin extract the rise of plasma insulin level was significantly suppressed after 60 min of administration. The plasma antioxidant activity measured by FRAP and ABTS assays increased in all treatments, but subjects treated with blue pea flower anthocyanins with or without sucrose demonstrated a significantly higher plasma antioxidant activity. Ingestion of blue pea flower anthocyanin extract with or without sucrose showed reduced levels of plasma malondialdehyde (MDA) during the postprandial period that indicating a low level of lipid peroxidation. The plasma thiol concentration which is an indicator of plasma antioxidant defence mechanism reduced significantly within 30 min after ingestion of sucrose but administration of blue pea flower anthocyanin extract with or without sucrose increased the plasma thiol level indicating the strengthening of plasma antioxidant defence mechanism by blue pea flower anthocyanins (Chusak et al., 2018). Therefore, blue pea flower anthocyanins could reduce the serum glycaemic index, MDA level and increase the plasma antioxidant level during the postprandial period. The application of blue pea flowers anthocyanins in food as a food colourant or functional food ingredient may provide these health benefits to consumers.

## Applications of Anthocyanins From Blue Pea Flower

Several studies have been done on the application of blue pea flower anthocyanins in many areas such as developing dye-sensitised solar cells, pharmaceuticals etc. (Gokilamani et al., 2013; Nair et al., 2015). This review focuses on the current research on the application of anthocyanins from blue pea flowers in the food industry. Several studies have been carried out on the application of anthocyanins from blue pea flowers as a natural food colouring agent and those food demonstrate antioxidant activity and bio-preservative properties (Table 3). A study was carried out to develop a functional beverage using blue pea flower extract, stevia extract and lime. Out of the three formulations screened from preliminary tests, the most acceptable beverage selected from the sensory evaluation had the combination of the above three constituents in ratio 983.25:1.75:15 in manufacturing 1 L of the beverage. The beverage developed with the above-mentioned combination of constituents were again tested with a sensory evaluation using a 9-point hedonic scale and for all the attributes (colour, sweetness, lime flavour, aroma and overall acceptability), a median score of 7 was obtained indicating moderate likeness. The antioxidant property of the functional beverage measured with DPPH, ABTS, FRAP and oxygen radical absorbance capacity (ORAC) assays are shown in Table 3. The total phenolic content of the functional beverage was 85.5 mg gallic acid equivalent/L. The beverage could be preserved at room temperature for 28 days without using any preservatives. When the pH of the beverage was adjusted between pH 2 to pH 4 to investigate the variation of colour, intense blue colour was observed between pH 3.5 and pH 4 (Lakshan et al., 2019). The reason for preserving the ability of blue pea flower extract incorporated functional beverage could be explained by the anti-microbial effect demonstrated by blue pea flower anthocyanins (Yanti et al., 2018). Therefore, blue pea flower anthocyanins can be used as a blue colourant in beverages having acidic pH between 3 to 4. Similar work was done by Marpaung et al. (2020) in which a crystallised functional drink powder was prepared with an anthocyanin-rich blue pea flower extract and supersaturated sugar solution and citric acid. The best formulation was selected using an sensory evaluation with a 9-hedonic scale and the formulation with 58 g sucrose, 0.46 g citric acid and 80 g of blue pea flower extract in 250 mL distilled water was selected as the most acceptable drink for colour, aroma, taste and overall acceptability. The initial TAC of the powder was reduced by 50% within 28 days when stored at 27°C in dark and the half-life of the anthocyanins was further reduced at high storage temperature. Interestingly, the initial antioxidant capacity (35–40% scavenging in DPPH radical scavenging activity) of the powder remained almost unchanged during the storage period of four weeks. Therefore, it would be better to use anthocyanins from blue pea flowers as a natural colouring agent for functional beverages with shorter shelf life.

**TABLE 3:** Application of blue pea flower anthocyanins as a natural colouring agent and their antioxidant/bioactivity.

Product	Antioxidant/Bioactivity	References
Functional beverage	DPPH radical scavenging activity – IC_50_ – 247.6 μL/mL ABTS radical scavenging activity – IC_50_ – 35.8 μL/mL FRAP – 14.9 mg Trolox equivalent/L ORAC – 122.2 mg Trolox equivalent/L	Lakshan et al., 2019
Functional drink powder	Powder showing 35–40% scavenging in DPPH radical scavenging activity	Marpaung et al., 2020
Yoghurt (liquid skim milk, UHT milk, pasteurised milk, UHT milk with skim powder, and pasteurised milk with skim powder)	69.3–437.04 ppm BHT equivalent in DPPH radical scavenging activity	Sutakwa et al., 2021
Muffin	Bactericidal effect	Ab Rashid et al., 2021

Sutakwa et al. (2021) investigated the antioxidant activity of yoghurts prepared with 10% (v/v) blue pea flower anthocyanin extracts. Five different types of milk (liquid skim milk, ultra-heat treated (UHT) milk, pasteurised milk, UHT milk with skim milk, and pasteurised milk with skim milk) were used to prepare the yoghurt. The antioxidant activity of yoghurt samples coloured with blue pea flower anthocyanins was significantly higher compared to the control samples and yoghurts prepared with skim milk showed the highest antioxidant activity (437.04 ppm measured by DPPH radical scavenging activity calculated using a standard linear equation with the butylated hydroxytoluene (BHT) as a standard curve). These studies evidently showed that the antioxidant activity of food increases when anthocyanins from blue pea flowers are used as a food colourant.

Thanh et al. (2020) investigated the application of blue pea flower anthocyanin extract in cupcakes. After baking at 170°C for 20 min, only 41.8% of the initial anthocyanin content was retained in the cupcake. The blue colour of the blue pea flower anthocyanin extract turned to greenish colour due to the pH change that happened in the dough. However, in the sensory evaluation, it was found that the aroma, colour, flavour and overall acceptability of the cupcakes with blue pea flower anthocyanins were higher compared with the control samples. The reason for the lower retention percentage could be attributed to the thermal deterioration of anthocyanins at high baking temperatures.

Studies show that the protection of anthocyanins with wall material by microencapsulation could enhance the stability of anthocyanins (Marsin et al., 2020). Therefore, the application of encapsulated blue pea flower anthocyanins in functional food provides a solution to enhance their stability in functional food. The application of encapsulated blue pea flower anthocyanins as a food colourant in baked food products (muffins) was studied (Ab Rashid et al., 2021). This study reported that the bacterial load on muffins with blue pea flower anthocyanins was significantly lower compared with the control sample. Therefore, blue pea flower anthocyanins have played two roles: one as a natural colouring agent and the other as a bio-preservative in this baked food product. This bio-preservative action of blue pea flower anthocyanins is supported by the study of Leong et al. (2017) where the anthocyanins from blue pea flower showed anti-fungal properties against the food-borne *Penicillium expansum* conidia. Nikijuluw and Andarwulan (2013) applied blue pea flower anthocyanin extracts to colour a yoghurt drink and rice. When the blue pea flower anthocyanin extract was added to yoghurt drink (pH 4.5) at a concentration of 3.37 × 10^–5^ mg anthocyanin/mL and rice (pH 7) at a concentration of 1.6 × 10^–4^ mg anthocyanin/g, the yoghurt drink has a purplish-blue colour and the rice had a dark blue colour. In this study, Brilliant Blue, a synthetic colourant was also added to yoghurt drink at a concentration of 3.13 × 10^–4^ mg/mL and to rice at a concentration of 3.2 × 10^–4^ mg/g and the colour of yoghurt drink and rice were green-blue and light blue, respectively (Nikijuluw and Andarwulan, 2013). Even though this study compared the stability of blue pea flower anthocyanin extract and Brilliant Blue, they did not investigate the stability of the colours after applying them in the food systems. It would be useful if a comparison was done in food systems to determine the colour stability and the bioactivity after adding the colourants.

The use of a colourimetric indicator in intelligent packaging has been used as a freshness indicator of perishable food products such as fish and meat. Colourimetric indicators provide real-time information on the freshness of the food material based on the pH dependant colour change (Priyadarshi et al., 2021). Microbial action on fish and meat produces chemical compounds such as amines that cause a pH change (Fletcher et al., 2018). Generally used chemical reagents as colourimetric indicators in intelligent packaging contain synthetic chemical compounds such as bromophenol blue and chlorophenol red are not safe to use for food packaging due to the possibility of migration of these compounds to food and their possible harmful effects on human health (Zhang et al., 2014; Poyatos-Racionero et al., 2018). Therefore, the invention of safer alternative reagents for this purpose is desired. The pH dependant colour variation of anthocyanins could be used to develop these kinds of intelligent packaging systems.

Several studies have been done on the application of anthocyanins from blue pea flowers as a colour indicator in intelligent packaging (Ahmad et al., 2020; Netramai et al., 2020; Salacheep et al., 2020; Singh et al., 2021). One study used anthocyanins from blue pea flowers in an intelligent packaging system incorporated in a film made with distilled water and 5% (w/v) sago powder (Ahmad et al., 2020). It was observed the colour of the packaging film changed from blue to green in 24 h when the chicken sample was kept at room temperature but the chicken sample kept in frozen condition did not alter the colour of the indicator even after 48 h (Ahmad et al., 2020). This study reported that the colour change of anthocyanin extract from blue pea flower at varying pH was more distinct compared with those of anthocyanin extracts from hibiscus, purple sweet potato and red yeast rice. Another study used anthocyanin extract from blue pea flowers as a colour indicator in gelatin film (Rawdkuen et al., 2020). A packaging film was made with gelatin, glycerol and anthocyanin extract from blue pea flower. The initial pH of gelatin film was 6 and the colour was blue. When pH reduced to 4, the film turned to violet and when pH increased to pH 8 the film turned to green. The incorporation of anthocyanin extract from the blue pea flower slightly reduced the tensile strength and water vapour permeability of the gelatin film but significantly increased the antioxidant capacity of the gelatin film (Rawdkuen et al., 2020). Another study used a gelatin film with blue pea flower anthocyanins as a colour indicating package to monitor fish freshness. Initially, the packaging film was dark bluish-purple at the beginning and turned to bluish-green when the fish was kept at room temperature for 24 h (Salacheep et al., 2020). Another study used anthocyanin extract from blue pea flowers in a freshness monitoring packaging for prawns made with starch and TiO_2_. The colour of the colour indicator changed from pink to green when prawns were stored at 4°C for 6 days (Mary et al., 2020). Similarly, Wu et al. (2021) developed an intelligent packaging film using gellan gum adding blue pea flower anthocyanin extract to monitor the freshness of shrimp. The colour of the film changed distinctly from blue to green when the shrimp were kept at 25°C for 24 h. The pH of shrimp changed from pH 5 to pH 8 within 24 h due to the production of nitrogenous compounds produced during spoilage. Therefore, blue pea flower anthocyanin extracts can be used to develop intelligent packaging systems to monitor the freshness of seafood as well.

In addition to freshness monitoring systems of animal products, Singh et al. (2021) used anthocyanins of blue pea flowers to develop an intelligent film to monitor the freshness of beverages. Anthocyanin extracts from *Clitoria ternatea* and *Carissa carandas* were incorporated into chitosan-poly (vinyl alcohol) films separately and used to monitor the freshness of milk and fresh orange juice. It was observed that incorporation of anthocyanin extracts did not significantly alter the physical properties of the film and the film with *Clitoria ternatea* anthocyanin extract was more pH-sensitive in terms of colour change compared with the film with the anthocyanin extract of *Carissa carandas*. The film with *Clitoria ternatea* anthocyanin extract showed a distinct change in colour for both milk and juice stored at 25°C after 72 h where the pH of milk changed from 6.2 to 4.1 and the pH of juice changed from 4.2 to 3.4 (Singh et al., 2021). Therefore, anthocyanins from blue pea flowers could be considered as a promising colour indicator for intelligent packaging.

## Conclusion and Future Perspective of Research

*Clitoria ternatea* or blue pea flower is an edible flower with medicinal and ornamental value. The blue pea flower is a rich source of polyacylated anthocyanins demonstrating higher stability than non-acylated anthocyanins. Blue pea flower anthocyanins demonstrate an intense and stable blue colour in acidic medium which facilitate their application in acidic food systems as a blue food colouring agent. The cellular and *in vitro* antioxidant activities of blue pea flower anthocyanins show their potential application in functional foods. Several studies have been conducted on investigating the application of blue pea flower anthocyanins as a food colourant in bakery products, yoghurt and functional beverages. Further research should be carried out on the application of blue pea flower anthocyanins in other food systems. Studies have shown antioxidant and antimicrobial activities of blue pea flower anthocyanins in different applications. Further research could be carried out to investigate the bioavailability and other functional properties of blue pea flower anthocyanins. Since there are limited blue food colourants available, blue pea flower anthocyanins will be a good alternative to be used as a natural blue food colouring agent.

## Author Contributions

GCVG: responsible for writing and editing the manuscript. YYL: responsible for reviewing and editing the manuscript. WSC: responsible for the conceptualisation, funding acquisition, and reviewing and editing of the manuscript. All authors contributed to the article and approved the submitted version.

## Conflict of Interest

The authors declare that the research was conducted in the absence of any commercial or financial relationships that could be construed as a potential conflict of interest.

## Publisher’s Note

All claims expressed in this article are solely those of the authors and do not necessarily represent those of their affiliated organizations, or those of the publisher, the editors and the reviewers. Any product that may be evaluated in this article, or claim that may be made by its manufacturer, is not guaranteed or endorsed by the publisher.
